# Intimate Partner Violence and Mental Health during Lockdown of the COVID-19 Pandemic

**DOI:** 10.3390/ijerph19052535

**Published:** 2022-02-22

**Authors:** Fabienne Glowacz, Amandine Dziewa, Emilie Schmits

**Affiliations:** Department of Psychology-Adaptation Resilience and Change Research Unit (ARCh), University of Liege-Place des Orateurs, 1-4000 Liège, Belgium; amandine.dziewa@uliege.be (A.D.); emilie.schmits@uliege.be (E.S.)

**Keywords:** intimate partner violence, COVID-19 pandemic, mental health

## Abstract

Background: This study took place in the context of the COVID-19 pandemic. The present research assesses the association between lockdown conditions (such as time spent at home, living environment, proximity to contamination and social contacts), mental health (including intolerance of uncertainty, anxiety and depression) and intimate partner violence within the community. This study evaluates the indirect effect of anxiety and depression on the relationship between intolerance of uncertainty and intimate partner violence (physical assault and psychological aggression). Methods: 1532 adults (80.8% of women, Mage = 35.34) were recruited from the Belgian general population through an online self-report questionnaire completed during the lockdown (from April 17 to 1 May 2020). Results: The results demonstrate that the prevalence of physical assault (including both perpetration and victimization) was significantly higher in men, whereas the prevalence of psychological aggression was significantly higher in women. Men reported significantly more violence during lockdown. Women, on the other hand, were more anxious and more intolerant of uncertainty. No difference between men and women was found for depression. Anxiety and depression significantly mediated the relationship between intolerance of uncertainty and physical assault and psychological aggression. Sex did not moderate the mediation. Conclusion: Clinical implications for public health policy are highlighted.

## 1. Introduction

On 11 March 2020, the WHO declared the COVID-19 outbreak a pandemic as the virus spread worldwide. One of the disturbing features of an emerging epidemic is that, as long as the precise cause and evolution are unknown, the uncertainty engendered by the situation can increase the level of psychosocial morbidity [1,2,3,4,5,6]. In an attempt to control this pandemic, governments across the world have taken action through restrictive measures unprecedented in the history of public health, such as lockdowns, social distancing and voluntary self-isolation [7,8,9,10]. These restrictive confinement measures put in place to counter the virus’ spread could have major consequences for mental health. Indeed, several studies conducted in China and in Europe have reported high levels of depressive and anxiety symptoms as well as poor sleep quality, and younger people have reported a significantly higher prevalence of generalized anxiety disorder and depression [7,9,10,11,12,13,14]. Furthermore, the restrictive measures are likely to increase the risk of family violence and reduce the options for support [15,16]. Being confined to one’s home can lead to tension and violence in couples where there was previously no violence, as well as increase the incidence of violence. In fact, social distancing and the orders to stay at home could lead to an increase in conflicts, disagreements and arguments due to the increased daily proximity of couples, but also by the limitation or absence of access to other social and public spaces (professional, recreational, sports, etc.) that contribute to the regulation of tensions and to the well-being of people. Moreover, other factors such as economic uncertainty, job loss, and being with children all the time may add to the stress experienced by both women and men, thereby increasing the risk of marital conflict and violence [17]. In addition, confinement may reinforce or facilitate control, surveillance and coercion strategies of perpetrators of intimate partner violence [18,19]. A number of experts have warned that women would be increasingly exposed to intimate partner violence (IPV) in a lockdown situation. In fact, a rise in reports of domestic abuse, in the number of calls from victims in distress, and demand for support has been noted in many countries. An increase in reports of domestic abuse, calls from distressed victims and requests for help was noted in Belgium where the “Ligne Écoute violences conjugales”, a domestic violence helpline, noted that the average number of calls a day tripled during the lockdown. Similarly, at the beginning of the lockdown, shelter services saw an rise in new requests, with an increase in up to +253% for the “Centre de prévention des violences conjugales et familiales” (one of the largest structures in Brussels). It began to decrease but remained at rates higher than pre-crisis levels (Brussels Council for Equality between Women and Men, 2021) [18,20].

In this way, the COVID-19 pandemic has revealed how IPV remains a major societal and health problem [16,21]. The risk of IPV was considered and guided public policy. Governments faced with the risk of IPV have encouraged either violence-reporting or the reception of victims during the lockdown. Pharmacies and grocery stores in France and Belgium have provided emergency warning systems to allow people to indicate that they are in danger and need support, using code words to alert staff [22,23].

While research on domestic violence prevention and treatment is ongoing and several sources have speculated on the impact COVID-19 has had on it [16,21,24], an increasing number of published peer-reviewed studies are analyzing IPV rates in light of the pandemic [25,26,27]. The main objective of this study is to assess, based on an online survey, the presence of IPV during the period of confinement, the associated factors related to lockdown and the pandemic and to individual mental health vulnerabilities.

### 1.1. Intimate Partner Violence

In recent years, intimate partner violence has been recognized as a real social and public health problem, and has become a central issue on the European political agenda. IPV includes acts of physical and sexual violence, emotional-psychological abuse, and controlling behaviors toward intimate partners of the same or opposite sex [28,29,30,31,32]. According to Johnson’s typology of IPV, there are two prevalent forms of IPV: intimate terrorism (IT) and situational couple violence (SCV). Intimate terrorism is part of a cyclical dynamic in which the abuser uses a variety of strategies (violent and non-violent) to control and terrorize the partner, including psychological, physical and sexual abuse, as well as intimidation and threats. The perpetrators of this violence (IT) are mostly men, which can be explained by the fact that it is rooted in patriarchy. Situational couple violence represents the violence that emerges when a conflict escalates into violence. While conflict is present in all couples, for some couples, these conflicts increase in frequency and intensity, culminating in the perpetration of violent acts [33,34]. The different studies have shown that situational couple violence is the most represented in general surveys, whereas intimate terrorism and violent resistance dominate in agency samples, and this is a source of difference across studies with respect to the gender symmetry of partner violence [35,36,37]. Our study focuses on the forms of both physical and psychological violence. Physical violence involves forceful physical contact that may vary from light pushes and slaps to severe beatings and lethal violence [31]. The term, psychological aggression, refers to acting in an offensive or degrading manner toward another, usually verbally, and may include threats, ridicule, withholding affection, and restrictions (e.g., social isolation, financial control). One of the most frequent forms of IPV in western societies is psychological. It can occur either in isolation or in conjunction with other forms of IPV and can be bidirectional [32,38,39,40,41].

Several studies and meta-analyses have identified poor mental health, including depression and anxiety, as one of the risk factors for physical and psychological violence, and it is associated with victimization and perpetration by both women and men [42,43,44,45,46]. However, this association may vary by gender and type of IPV [47]. Overall, symptoms of anxiety and depression were recognized as very high during the pandemic containment period [7,9,10,11,12]. Various studies have estimated the effects of the COVID-19 pandemic on mental health and have linked financial stress, food insecurity, fear of infection and increased time spent with a partner to increased stress. Stress, frustration and lack of control can exacerbate the psychopathological problems associated with IPV, further precipitating violent episodes. Situations that increase stress appear to be one of the important risk factors for victims of partner violence [26,48,49].

### 1.2. Intolerance of Uncertainty

In relation to the unpredictability of the future, the COVID-19 pandemic is globally dominated by significant uncertainties about the virus and how to control it and the variants [50]. In this context, distress related to uncertainty is an understandable, and even appropriate, reaction. However, if the threat and uncertainty become pervasive, it can disrupt the psychological and social functioning of the individual [4]. In this regard, intolerance of uncertainty (IU) refers to individual differences in the difficulties of coping with the experience of uncertainty. IU can result in a range of cognitive, emotional and behavioral responses aimed at avoiding and/or resolving the aversive experience [51,52]. Thus, the inability to deal with distress arising from uncertain situations can have a detrimental effect on mental health, leading to various psychopathological symptoms, such as anxiety or depression [53,54,55]. Very recent research has shown that COVID-19-related IU played a fundamental role in psychopathological symptoms (depression and anxiety) in the population during confinement [9,56,57]. IU may also be implicated in maladaptive externalizing behaviors and diagnoses [51]. Distress resulting from elevated IU could, in turn, increase the propensity for aggressive behavior in some individuals [58]. However, few studies have investigated the role of IU in aggressive behavior and violence, and none have done so in the highly anxiety-provoking context of a pandemic crisis. Nevertheless, one study has shown the links between IU and anger/aggression [59]. Moreover, the risk for the externalizing spectrum of psychopathology related to difficulties in tolerating uncertainty may lead individuals to engage in risky behaviors to alleviate distressing or unpleasant emotions [60]. No studies, to our knowledge, have dealt with IU and IPV. However, with feelings of uncertainty being particularly prevalent during an emerging pandemic, our study will integrate the variable IU in order to evaluate its links with violence between partners. Anxiety and depressive symptoms will be examined in our study as predictors of IPV, as well as a mediator, in particular, intolerance of uncertainty.

### 1.3. Our Study

Whilst carried out during the COVID-19 pandemic, the main purpose of this study is to first assess the (H1) association between “proximal factors” related to confinement, perpetration and victimization IPV within the community: lockdown conditions, such as time spent at home, the living environment, the frequency of social contact through digital media, proximity to contamination, and the intolerance of uncertainty and mental health (anxiety and depression). Second, (H2) the roles that intolerance of uncertainty, anxiety and depression have played in the increase in perpetration and victimization IPV cases during the pandemic crisis will be considered, as well as the gender dimension (H3), as the suggested associations should be gender-related.

**Figure 1:**
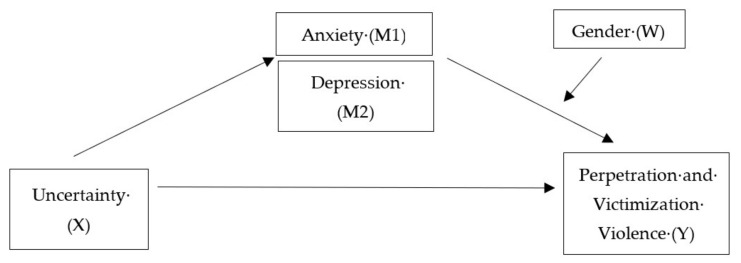
The proposed models. Note. Distinct models have been tested: the indirect effect of anxiety (M1) and depression (M2) in the relationship between intolerance of uncertainty (X) and physical (model 1) and psychological (model 2) violence (Y), according to gender (W) (model 3 and model 4).

Finally, as the literature highlights that intolerance of uncertainty could implicate anxiety and depressive disorders [2,61,62,63] and that these mental health problems could be risk factors for violence [42,43,44,45,46], the present study also proposes to assess the indirect effects of anxiety and depression on the relationship between intolerance of uncertainty and intimate partner violence (including both victimization and perpetration). (H4) Mental health-related variables mediate the predictive effect of IU on perpetration and victimization IPV (mediation model) (H5) differently among men and women (moderated mediation model). Physical assault and psychological aggression will also be considered separately. Models are illustrated in Figure 1.

## 2. Methods

### 2.1. Participants

A total of 1532 adults were recruited from the general population through an online self-report questionnaire. All participants were engaged in a romantic relationship and lived together.

### 2.2. Data Collection Procedure

This research consisted of a web-based electronic survey. Invitations to participate were broadly and non-selectively sent by email to the general population (e.g., personal and professional contacts and a mailing list), as well as posted on multiple online spaces (e.g., social and professional networks). Inclusion criteria consisted of being over 18 years, being engaged in a romantic relationship and living together. The questionnaire was developed in French. The experimental protocol complied with the Belgian guidelines for studies involving human beings and was approved by the IRB of the University of Liège. The data collection was conducted in accordance with the Helsinki Declaration. Data were obtained through an online self-report questionnaire distributed one month after the beginning—in other words, just before the end of the lockdown—during the period from 17 April to 1 May 2020. We complied with ethical research standards in providing information about the project and asking for consent to participate in the online survey. All respondents provided informed consent. In order to answer any question or to deal with any inconvenience caused by scales (feelings of discomfort, distress or danger), the researcher’s e-mail address was given at the beginning and the end of the questionnaire and a videoconference meeting with a specialized psychologist in the field of domestic violence was offered free of charge. None of the participants requested this assistance.

### 2.3. Materials and Measures

#### 2.3.1. Sociodemographic Data and Lockdown Conditions

These data are part of a large database collected during lockdown, related to the COVID-19 crisis [9]. It includes the usual sociodemographic data (gender, age, country of residence, educational background and marital status). Data on lockdown conditions were also collected through self-created questions: children and their presence during this period (dichotomous variables: Yes/No), professional situation during the lockdown (including four categories: student, working from home, usual workplace, no work), time spent at home (a score ranging from 0, working out of home during the day, to 2, at home without work all the day), living environment (a score ranging from one to eight, evaluating the surface area of the accommodation as well as the availability of a terrace and a garden) and loss of financial income (dichotomous variable: Yes/No). The frequency of social contact was also assessed through 7 items on a 4-point Likert scale (1 = never; 4 = everyday), evaluating contact with friends, family, colleagues, etc., through digital media. A high score indicated a high frequency of social contact through digital media. Primary (oneself) and secondary (a close person) coronavirus contaminations were specified (three modalities: not infected, infected but not tested, tested positive for the coronavirus). On this basis, a score of proximity to contamination was determined, including a gradual score from 0 (neither the person him/herself, nor a loved one, was infected) to 8 (the person him/herself and a loved one had tested positive for the coronavirus). A high score indicated a high level of proximity to contamination.

#### 2.3.2. Mental Health-Related Variables

Anxiety and depression were evaluated by the two subscales of the Hospital Anxiety and Depression scale (HAD) [64]. The HAD is a 14-item scale that proposes seven items related to anxiety (in the present sample, α = 0.80) and seven related to depression (in the present sample, α = 0.68), scoring from 0 to 3. Cut-off points of 8 and 11 were identified [65]. Only two items (“Uncertainty makes me uneasy, anxious, or stressed” and “When I am uncertain I can’t function very well”) of the validated Intolerance of Uncertainty Scale (IUS) [52] were included to evaluate reactions to uncertain situations (in the present sample, α = 0.72), scoring from 1 (not at all corresponding) to 5 (extremely corresponding). An additional item, also scoring from 1 (not at all corresponding) to 5 (extremely corresponding), was created, namely “Uncertainty makes me more aggressive with my partner in terms of my words, gestures and attitudes” (named “Uncertainty-related aggression”). Finally, a question evaluating if the person had consulted a psychologist (through a videoconferencing system) during the lockdown (dichotomous: Yes/No) was also included.

#### 2.3.3. Intimate Partner Related-Variables

The length of the romantic relationship was assessed through a question with 5 modalities (from less than 6 months to more than 20 years). The higher the score, the longer the relationship. The 20-question short-form Revised Conflict Tactic Scales (CTS2S) [66,67] was used to measure intimate partner violence. The instruction and quotation were adapted to the context of the lockdown period. Three types of IPV were identified (physical assault, psychological aggression and sexual coercion) as well as emotional and psychological negotiation. Only physical assault (e.g., pushing, kicking, beating: sprains and bruises) and psychological aggression (e.g., yelling, arguing, threatening harm and destroying belongings) in the relationship were considered in the present research (respectively, α = 0.73 and 0.63). An additional question (subjective self-evaluation) on the evolution of violence (violence in general, not a specific kind of violence) was included, with 4 modalities (no violence, decreased violence, continuing violence and increased violence).

### 2.4. Data Analysis

SPSS 26 software (IBM Corporation, Armonk, NY, USA) was used to perform the statistical analyses. We conducted (1) descriptive statistics to have an overview of demographic data, and perpetration and victimization violence; (2) Cronbach’s alpha to measure consistency reliability; (3) Spearman’s correlations to evaluate the links between all the variables; (4) Chi-square (with Phi coefficient) and Mann–Whitney U tests to compare men and women. Statistical significance was set at *p* < 0.05. To test the indirect effect of anxiety and depression on the relationship between intolerance of uncertainty, perpetration and victimization violence, (5) mediation models were conducted. To assess the effect of gender on these models, moderated mediation models were run. PROCESS modeling, as outlined by Preacher and Hayes], was applied and a bootstrapping method (10,000 bootstrap samples) was used. This method is a nonparametric approach to effect-size estimation and hypothesis testing that is not based on large-sample theory and, therefore, circumvents the power problem associated with asymmetries [68]. When zero is not included in the bootstrap confidence intervals, it is possible to set a significant indirect effect (mediator effect) or conditional effect (moderator effect)—reflected in the index of moderated mediation [69,70,71]—at *p* < 0.05. Tested models are shown in Figure 1.

## 3. Results

### 3.1. Descriptive Statistics and Correlations

Among the 1532 participants, 80.8% were women (*n* = 1238) and 19.2% were men (*n* = 294). No non-binary gender was identified. They were aged between 18 and 83 years (M = 35.94, SD = 14.84). The age ranges are similar for women (M = 35.25; SD = 14.21) and men (M = 38.86; SD = 17.03). Descriptive statistics concerning sociodemographic data and proximal factors related to the lockdown are described in Table 1. Note that more than half of couples have been together for more than 10 years, almost half live with their children and nearly half have worked from home during the lockdown.

The first results show prevalence rates concerning intimate partner violence. Among the subsample of perpetrators of intimate partner violence (33.4% of the total sample, *n* = 511), 84.5% (*n* = 432) are also victims of violence. Among the subsample of victims of intimate partner violence (28.2% of the total sample, *n* = 432), 100% are also perpetrators. In other words, among the total sample (*n* = 1532), 28.2% are both perpetrators and victims (*n* = 432), 5.2% are only perpetrators (*n* = 79), and 0% are only victims. On the basis of these results, future analyses will not focus on the perpetrators/victims’ perspective, but on the form of severity and frequency of violence, especially physical assault and psychological aggression. Considering each item of the CTS2S separately, the prevalence rates are presented in Table 2 for minor (M) and severe (S) physical assault and psychological aggression. As violence should be gender-related, the prevalence are presented for the total sample and women/men subsamples.

Second, lockdown-related, mental health and intimate partner variables are considered. Means and standard deviations for proximity to contamination, living environment, time at home, social contacts, couple duration, uncertainty-related aggression, anxiety, depression, intolerance of uncertainty, physical assault and psychological aggression, as well as Spearman’s correlations between these variables, are shown in Table 3.

Physical assault and psychological aggression (including both perpetration and victimization) were significantly positively related to aggression associated with uncertainty, anxiety, depression, and intolerance of uncertainty (H2 confirmed), and significantly negatively associated with living environment and couple duration. Only psychological aggression was positively associated with proximity of contamination (H1 partially confirmed).

### 3.2. Group Comparisons

The prevalence of physical assault (including both perpetration and victimization) was significantly higher in men, whereas the prevalence of psychological aggression (including both perpetration and victimization) was significantly higher in women. A total of 3.6% of the sample reported an increase in violence during confinement. Of these, 45% had never experienced violence, and 55% had experienced violence before the confinement. Compared to women, more men reported an increase in violence, most of whom had experienced it before the confinement. Women were found to be more anxious and more intolerant of uncertainty. No difference between men and women was found for depression (Table 4) (H3 partially confirmed).

Additionally, differences between participants living with children during confinement and those who were not have been assessed. The results demonstrated that both groups did not differ concerning anxiety (U = 301,140.00, *p* = 0.29), depression (U = 294,049.00, *p* = 0.81), intolerance of uncertainty (U = 279,033.50, *p* = 0.12), physical (U = 290,379.50, *p* = 0.75) and psychological (U = 303,254.00, *p* = 0.11) violence (including both perpetration and victimization). Therefore, this variable was not included in the following models.

### 3.3. Mediation Models

To test the indirect effect of anxiety (M1) and depression (M2) in the relationship between intolerance of uncertainty (X), physical assault (model 1) and psychological aggression (model 2) (Y), mediation models with multiple mediators were conducted (model 4 of PROCESS macro) [70,71]. As shown in Table 5, anxiety and depression significantly mediated the relationship between intolerance of uncertainty, physical assault and psychological aggression (including both perpetration and victimization). Because intolerance of uncertainty no longer affected perpetration and victimization violence after mediators were included, both mediations were complete (H4 confirmed).

### 3.4. Moderated Mediation Models

As a gender difference was demonstrated for many variables, it was included in the model. To test the indirect effect of intolerance of uncertainty (IV) on physical assault and psychological aggression (DV) through anxiety and depression (M) moderated by gender (W), moderated mediation (moderator x mediator interaction) models with multiple mediators were conducted (model 14 of PROCESS macro) [70,71]. Our results demonstrate that gender does not significantly moderate the mediation models for either model: (model 3) the indirect effect of intolerance of uncertainty to physical assault through anxiety (index = 0.01, BootSE = 0.02, BootLLCI = −0.03, BootULCI = 0.07), the indirect effect of intolerance of uncertainty to physical assault through depression (index = 0.01, BootSE = 0.02, BootLLCI = −0.01, BootULCI = 0.06), (model 4) the indirect effect of intolerance of uncertainty to psychological aggression through anxiety (index = 0.01, BootSE = 0.03, BootLLCI = −0.05, BootULCI = 0.08), and the indirect effect of intolerance of uncertainty to psychological aggression through depression (index = 0.01, BootSE = 0.02, BootLLCI = −0.04, BootULCI = 0.05). Therefore, anxiety and depression completely mediate the relationship between intolerance of uncertainty, physical assault and psychological aggression (including both perpetration and victimization) for both men and women (H5 not confirmed).

## 4. Discussion

Since containment measures related to the COVID-19 crisis were announced in March in Europe and Canada, an increasing number of published peer-reviewed studies are analyzing IPV rates in light of the pandemic [25,26,27]. Our study is one of the early studies that evaluated perpetration and victimization violence between partners during confinement in the community to assess the association between lockdown conditions and mental health, allowing us to identify avenues for intervention and prevention.

During the confinement period, 33% of the participants had experienced at least one form of psychological or physical violence within their couples after 4 weeks, without taking sexual violence into account. Few data are available in Belgium for the period before the crisis; however, in 2010, the Institute for the Equality of Women and Men estimated that one woman in seven had been confronted with at least one act of violence committed by her (ex-)partner in the previous 12 months, and one couple in eight was confronted with psychological violence. Our study reports a higher prevalence. However, the percentage of severe physical and psychological violence cases among participants in our study was very low (victimization of severe physical assault (punched, kicked or beaten by partner) was 0.5% and victimization of severe psychological aggression was 2%). These results represent only one facet of perpetration and victimization violence in couples during the COVID-19 pandemic. Indeed, when studying violence in couples, it is important to consider the methods of population recruitment, the method of violence assessment and the population studied. Different types and degrees of IPV severity (intimate terrorism and situational partner violence) may be more prevalent depending on the sample type [33]. Our online study conducted during containment within the community allowed for rapid recruitment from the entire population, including a large amount of variability, which made it ideal for studying IPV. However, the online study of non-clinical samples tended to identify minor violence in couples, and more often situational and bidirectional violence, especially with the CTS2, which considers victimization and perpetration for all items. As a type of selection bias, perpetrators and victims of intimate terrorism-type violence and more severe violence may be less likely to respond to these online surveys, as victims may be prohibited from responding and perpetrators may be unlikely to report their own actions. Access to these surveys require the use of clinical samples distributed in shelters, hospitals or among the police, which was not possible during the period of confinement [36]. According to extensive studies, our results reflect that 84.5% of perpetrators were also victims of violence, and among victims of IPV, all were perpetrators. These associations may reflect the use of psychological and physical aggression in the context of situational couple altercations [30,32]. Our data do not allow us to determine the context, intentions and reactions of the perpetrator and victim partners, and although our results seem to reflect situational violence, some of it may be part of intimate terrorism-type violence. Studies based on qualitative approaches using interviews are needed to look deeper into the types of violence experienced during periods of confinement and deconfinement in the COVID-19 pandemic.

It should be noted that women are significantly more involved in psychological assaults, and men in physical assaults. However, men reported that involvement in IPV significantly increased during the lockdown. It is possible that the context of confinement may have increased the amount of time spent at home, increased tensions, conflict and violence for men, but it is also possible that men may have identified and experienced these experiences as violence because of the confinement when it may have appeared trivialized/normalized before. Previous studies have shown that an increase in the intensity and seriousness of violence can lead a victim to become aware of the violence [72,73]. Our results would suggest that the fact of being confronted with violence for men and being confined at home and in couples without other social living spaces could lead to an identification and awareness of this violence. Additionally, those in the youngest relationships are likely to experience physical and psychological violence (including both perpetration and victimization) during confinement, as well as to present symptoms of psychological distress (anxiety and depressive symptoms) associated with such violence, and intolerance of uncertainty perceived as generating, among other things, aggressiveness towards the partner (see correlations in Table 2). These findings highlight that young couples are more at risk in terms of physical and psychological violence, a finding already highlighted in previous research [74], and psychological distress in confinement [11]. Couples in older relationships may be better able to withstand confinement and provide supportive and safe environments for their partners to cope with the stress and uncertainty generated by the pandemic and confinement.

This novel situation of confinement during the COVID-19 pandemic has locked families and couples within their homes, thereby increasing the amount of time couples spend together on a daily basis, while decreasing the possibilities for contact and social relations with the outside world. Although, our data highlighted that social contact through digital media should be a risk factor for uncertainty-related aggression and anxiety, but a protective factor for depressive symptoms. Interestingly, our study also shows that work at home or absence from work, therefore, being more present at home, is significantly associated with depressive symptoms, but is not correlated with intimate partner violence. In contrast, the results reveal that physical and psychological violence is associated with smaller living spaces that lack a garden or terrace. Since housing is an indicator of a person’s socioeconomic level, it can be assumed that socially disadvantaged couples have been more affected by confinement. Indeed, a smaller living environment is also associated with the presence of anxiety, depression, intolerance of uncertainty and aggression related to uncertainty (see correlations). Although it is certainly the case that middle class and affluent families also experience cases of domestic violence, studies consistently indicate that as the financial status of a family increases, the likelihood of domestic violence decreases [75,76]. Couples experiencing financial and family stressors during the pandemic are likely to have an increase in the number of arguments, conflicts, and common couple violence during sustained social isolation and physical proximity, particularly among young and newly formed intimate relationships [17,26]. Sharma and Borah (2020) consider that the increase in domestic violence during the COVID-19 pandemic is an indirect driver of economic and social crisis [77].

The psychological impact of containment and the COVID-19 pandemic crisis on the mental health of the population is now demonstrated, with higher rates of anxiety and depression observed, which are linked to, among other things, IU [7,11,12,78]. Pandemic crises sow uncertainty which can last for a long time; intolerance of uncertainty is a risk factor for the mental health of the population [3,4]. Furthermore, poor mental health including anxiety and depression is a risk factor for intimate partner violence (IPV), perpetration and victimization [42,43,44,45,46] among men and women.

Recent research suggests that IU may be an important contributor to anxiety disorders and depression symptoms. This is the first study to examine whether IU predicts intimate partner violence (including both perpetration and victimization) in the context of confinement, and if mental health (anxiety and depression) could explicate and participate to this relationship. Interestingly, our study demonstrates that intolerance of uncertainty predicts the perpetration and victimization of physical and psychological violence in confined couples by increasing depressive and anxiety symptoms for both men and women. Individuals who are intolerant of uncertainty are more at risk of feeling anxious or depressed and are, therefore, more likely to experience IPV. IU alone does not appear to explain by itself the increase in the rates of this type of violence. Rather, it seems to be because individuals experience or are vulnerable to anxiety and depression related to uncertainty that rates of intimate partner physical and psychological violence increase (complete mediation). This phenomenon would be relevant for both women and men (non-significant moderated mediation). These major findings are a reminder that depression can increase the risk of violence and involvement in verbal conflicts [79]. They also highlight that depression and anxiety for both men and women are real risk factors for physical and psychological violence during the confinement.

Our study, conducted during a pandemic lockdown, incorporated intolerance of uncertainty into models of psychological and physical violence (including both perpetration and victimization). Deconfinement will give rise to even more uncertainty than the confinement period, as subjects will no longer be required to remain in their homes and the rules on social interaction patterns and risks will be less clear. It is, therefore, important to incorporate this variable into our psychological models. Our results show, however, that reducing uncertainty will not directly decrease the risk of violence, nor will it directly increase it, but for subjects in whom uncertainty is anxiety-inducing and depressogenic, intolerance of uncertainty may increase and/or lead to violence.

These results underscore the importance of paying attention to the mental health of individuals in studies of IPV, especially during periods of confinement and deconfinement. This is especially significant if individuals are suffering from depression as it is a factor of recidivism [80]. Our study has made it possible to highlight the existence of physical and psychological IPV, which can affect both women and men living in intimate relationships. While public policies during confinement have focused on violence against women and on severe violence, our survey highlights the need to also take into account minor violence within couples, and violence perpetrated and suffered by women and men [81]. IPV in times of crisis and the associated mental health factors require a combination of social, medical and legal responses. As informal contacts are the main detection and support system, community-based initiatives and public media should be used to raise awareness of the increased risk of IPV during the pandemic [21,82]. Governments need to do more to alert the public to additional stressors and communicate about coping strategies [27]. A proactive approach focusing on well-being, hopefulness or self-efficacy can be useful during the COVID-19 period and help reduce the social crisis [77]. Given the impact that the pandemic may have on mental health and the highlighted links to partner violence, health professionals need to pay particular attention to the mental stability of their patients, intervening to reduce the exacerbation of co-morbid psychiatric disorders and, thus, reduce the potential risk of violence [82]. In a time of reaction, developing protocols and training frontline professionals (police officers, psychologists, doctors, etc.) in IPV screening procedures and mental health risk factors in times of crisis could help to better identify people at risk [49,83].

## 5. Strength and Limitations

The present research has several strengths, such as the large sample, recruitment across several countries, the diversity and completeness of studied variables, and the solidity and relevance of tested statistical models. This is one of the first studies to evaluate intimate partner violence (including both perpetration and victimization), including variables associated with confinement conditions, depression, anxiety and intolerance to uncertainty, and highlighting an innovative model. The first limitation is that women are overrepresented. Gender has been taken into account in all analyses to neutralize this problem. Additionally, we used a non-validated 2-item version of the IU scale. Another limitation is that, as mentioned by Kaukinen [17], the samples from self-report data may not include all women who are victims of the most severe types of IPV or those who are victims of the COVID-19 disease. Finally, our study evaluated psychological and physical violence on the basis of CTS2, which does not measure the consequences or the causes of the assault (such as the desire to dominate), or dynamics of violence. Although the CTS2 could be criticized for being reductionist in its sole focus on the presence of an act, and ignoring the context in which the act took place, it does afford measurement of the form, severity and frequency of different acts in our study “during lockdown” [84]. Future research may need careful joint analyses of self-report survey data, estimates from law enforcement agencies and clinical data during and after COVID-19. This will allow us to tap into diverse types of intimate partner abuse and explore the way in which COVID-19 disease progression may place women and men at further risk for physical violence, emotional and financial abuse, and coercive control [17]. Future research should also consider sexual coercion during lockdown, and interpret IPV through aggressive/violent behavior theories. Subsamples should also be required from vulnerable/precarious populations such as those in shelters, hospitals and from the police, namely populations with little access to online surveys. Future research is needed to highlight the intimate partner violence process in at-risk populations, especially mental health-related and crisis-associated risks to develop intervention and assistance strategies during crisis and post-crisis periods, such as the one we are experiencing with COVID-19.

## 6. Conclusions

During the COVID-19 epidemic, people faced a sense of uncertainty that affected levels of anxiety and depression, and the risk of IPV. First, clear and consistent information regarding the disease and management plan should be provided to everyone to avoid panic, confusion and to reduce uncertainty [85]. Secondly, there is a need for programs to prevent IPV of varying severity among youth, men and women, and especially among those who are psychologically fragile in the context of the uncertainty caused by the pandemic, and particularly for socially and psychologically vulnerable people. In addition, it is important to strengthen psychological first aid; it is a crucial early intervention that focuses on the mental health of the population by providing psychosocial support during outbreaks such as COVID-19 [85,86]. It is also necessary to make all front-line medical and psychological professionals aware of the risk of IPV so that they integrate it into their assessment and interventions with patients. These early interventions could offer help to both victims and perpetrators and stop the escalation and/or installation of more severe IPV. Finally, it seems appropriate to continue these interventions after confinement.

## Figures and Tables

**Table 1:** Demographic data and factors related to the lockdown.

		*%*	*n*
Country	Belgium Another French-speaking country	82.6 17.4	1265 266
Education	Higher Education Others	80.1 19.9	1225 306
Relationship	Heterosexual Same-sex	96.2 3.8	1473 58
Duration of the relationship	Less than 6 months Between 6 months and 2 years Between 2 and 10 years Between 10 and 20 years More than 20 years	2.1 11.1 35.6 25.5 25.7	32 170 545 391 394
Children	Yes, and living with them Yes, but not living with them No	46.7 11.3 42	715 173 643
Work	Working from home Taking time off or out of work Working at their workplace Students	46.4 25.1 14.9 13.6	711 384 229 208
Lost of financial income	Yes No	23.8 76.2	364 1167
Infection to coronavirus (themselves)	No Yes maybe, but not tested Yes, tested positive	86.9 12.5 0.6	1331 191 10
Infection to coronavirus (one person close to them)	No Yes maybe, but not tested Yes, tested positive	69.1 19.6 11.3	1059 300 173
Consultation with a psychologist during the lockdown	Yes No	6.3 93.7	96 1435

**Table 2:** Prevalence for each item of physical assault and psychological aggression subscales.

	Total (*n* = 1532)		Women (*n* = 1238)		Men (*n* = 294)	
**Physical assault**	*%*	*N*	*%*	*N*	*%*	*N*
Victim—Sprain, bruise or small cut (M)	6.8	104	6.5	81	7.8	23
Perpetrator—Sprain, bruise or small cut (M)	5.8	88	4.9	62	8.9	26
Perpetrator—To push, shove or slap (M)	1.8	28	1.9	24	1.4	4
Victim—Pushed, shoved or slapped (M)	2.3	35	1.9	24	3.8	11
Perpetrator—To punch, kick or beat-up (S)	0.3	5	0.2	3	0.7	2
Victim—Punched, kicked or beat-up (S)	0.5	8	0.2	4	1.4	4
Victim—To need to see a doctor (S)	0.4	6	0.3	4	0.7	2
Perpetrator—To send to see a doctor (S)	0.5	8	0.3	4	1.4	4
**Psychological aggression**						
Perpetrator—To insult or swore or shout or yell (M)	30	460	31.8	394	22.5	66
Victim—Insulted, swore or shouted or yelled (M)	27.5	421	28	347	25.3	74
Perpetrator—To destroyed something (S)	0.9	14	0.9	12	0.7	2
Victim—Have something destroyed (S)	1.1	17	1.0	13	1.4	4

**Table 3:** Spearman’s correlations, descriptive statistics and internal consistency.

	1.	2.	3.	4.	5.	6.	7.	8.	9.	10.	11.	M	SD
**1. Prox.**	1											0.93	1.68
**2. Envi.**	−0.02	1										5.57	1.77
**3. Home**	−0.03	−0.01	1									1.10	0.62
**4. Cont.**	0.10 **	0.07 **	−0.05 *	1								16.49	3.76
**5. Couple**	−0.06 *	0.37 **	0.08 **	−0.04	1							3.62	1.04
**6. Agress.**	0.04	−0.07 **	0.01	0.06 *	−0.07 **	1						2.76	1.25
**7. Anx.**	0.04	−0.10 **	−0.03	0.10 **	−0.16 **	0.35 **	1					6.75	3.85
**8. Dep.**	0.02	−0.13 **	0.10 **	−0.07 **	−0.06 *	0.30 **	0.52 **	1				7.42	3.54
**9. Uncert.**	−0.01	−0.14 **	0.04	0.03	−0.15 **	0.49 **	0.50 **	0.40 **	1			6.62	1.97
**10. Phys.**	0.03	−0.05 *	0.03	0.01	−0.08 **	0.20 **	0.14 **	0.16 **	0.15 **	1		8.25	1.05
**11. Psycho.**	0.06 *	−0.09 **	0.03	0.04	−0.11 **	0.34 **	0.20 **	0.22 **	0.16 **	0.31 **	1	4.83	1.44

Note: Prox. = proximity to contamination. Envi. = living environment. Home = time spent at home. Cont. = social contacts. Couple = couple duration. Agress. = uncertainty-related aggression. Anx. = Anxiety. Dep. = depression. Uncert. = intolerence to uncertainty. Phys. = physical assault. Psycho. = psychological aggression. * *p* < 0.05. ** *p* < 0.001.

**Table 4:** Prevalence and gender differences of intimate partner violence behaviors (both perpetration and victimization).

	Total (*n* = 1532)		Women (*n* = 1238)		Men (*n* = 294)		X^2^ (dl = 1)	*p*	Phi
	***%***	***N***	***%***	***N***	***%***	***N***			
Physical assault	9.2	140	8.4	104	12.3	36	4.27	0.03	0.05
Psychological aggression	33.8	517	35.2	435	28.0	82	5.49	0.01	−0.06
Increased violence	3.6	55	2.8	35	6.8	20	19.38	0.001	0.11
	**M**	**SD**	**M**	**SD**	**M**	**SD**	**U**	***p***	
Anxiety	6.75	3.85	6.98	3.85	5.73	3.59	146,202	<0.001	
Depression	7.42	3.54	7.49	3.58	7.10	3.31	168,932	0.05	
Uncertainty	6.62	1.97	6.82	1.88	5.75	2.11	129,355	<0.001	

**Table 5:** Mediation Analyses with Multiple Mediators.

Variables		Effect	(Boot) *SE*	*t*	*p*	(Boot) LLCI	(Boot) ULCI
**Model 1**		**DV = Physical Assault**					
*y* = Physical Assault *x* = Uncertainty	Total effect of *x* on *y*	0.06	0.01	4.94	<0.001	0.0403	0.0933
	Direct effect of *x* on *y*	0.01	0.01	0.50	0.61	−0.0227	0.0384
m = All mediators	Indirect effect of *x* on *y*	0.06	0.01			0.0298	0.0958
m1 = Anxiety		0.03	0.01			0.0099	0.0629
m2 = Depression		0.02	0.01			0.0104	0.0414
**Model 2**		**DV = Psychological Aggression**					
*y* = Psychol Violence *x* = Uncertainty	Total effect of *x* on *y*	0.12	0.01	6.45	<0.001	0.0827	0.1548
	Direct effect of *x* on *y*	0.02	0.02	0.87	0.38	−0.0228	0.0596
m = All mediators	Indirect effect of *x* on *y*	0.10	0.01			0.0718	0.1312
m1 = Anxiety		0.05	0.01			0.0308	0.0891
m2 = Depression		0.04	0.01			0.0226	0.0602

Note: SE = Standard Error; LLCI = lower limit of confidence interval; ULCI = upper limit of confidence interval. Observations with missing values were removed from the analysis.

## Data Availability

The datasets used and/or analyzed during the current study are available from the corresponding author on reasonable request.
